# Correlations among Work Stressors, Work Stress Responses, and Subjective Well-Being of Civil Servants: Empirical Evidence from China

**Published:** 2019-06

**Authors:** Ling ZHANG, Jue FU, Benxian YAO, Yuesong ZHANG

**Affiliations:** 1. Center for Cultivation of Morals and Basic Law, Shaoxing University, Shaoxing, China; 2. College of Educational Science, Anhui Normal University, Wuhu, China; 3. College of Teacher Education, Hefei Normal University, Hefei, China

**Keywords:** Civil servants, Work stressors, Work stress responses, Subjective well-being

## Abstract

**Background:**

The work stress of civil servants has gradually increased as a result of the modernization of China’s national governance system and capacity. However, research on the correlations among work stressors, work stress responses, and subjective well-being (SWB) of civil servants is scarce.

**Methods:**

In accordance with the current research status on work stress and SWB, a survey of 874 civil servants in China was carried out from May to June 2018. The revised stress response questionnaire of civil servants, work stressors questionnaire of civil servants, and a simplified edition of the SWB scale of China’s urban residents were used in this study.

**Results:**

Superiors impose the major work pressure on civil servants, followed by interpersonal relationship, work particularity, career prospect, work task, perfectionism, and job responsibility. The work stressors of civil servants were significantly related to gender, age, marital status, working years, educational background, and position (*P*<0.05). The work stressors of civil servants were significantly positively correlated with work stress responses (*P*<0.05). The work stressors and work stress responses had a significantly negative correlation with SWB.

**Conclusion:**

SWB can be accurately predicted by work stressors and work stress responses. These findings can provide references and guidance for the society and government sectors to accurately understand and cope with the treatment of civil servants, formulate work stress management countermeasures, and create a high-level working environment for civil servants.

## Introduction

With the accelerating pace of life and intensifying industrial competition in the modern society, accidents caused by work stress happen occasionally. An increasing number of people are struggling with work stress. Among them, civil servants bear psychological pressure that brought about by the whole socio and cultural change to all groups and the reform of government organizations. Civil servants are professionals with a high risk of mental disorders (1). Relevant data show that, 33.8% of the civil servants were afflicted with high job strain (2). More than 50% of Japanese civil servants have received compensation for mental disorders since 2000. Approximately 47% of compensated mental disorders were caused by work stress. Policemen and fire department officers were the groups with the highest occurrence of mental disorders induced by work stress (3). The detection rate of depression among civil servants in Beijing was 18.2%, including 19.6% males and 16.2% females (4). With the deepening reform of the civil servant system in China and the implementation of job bidding, contract employment, and accountability systems, the work pressure of civil servants has gradually increased. Accordingly, the problems of psychological health of civil servants have attracted widespread social awareness.

Individuals who perceived pressure strongly make strong responses to stress and easily develop psychical discomfort and negative emotional responses (5). The stress response was significantly correlated with psychological health (6). Work stress has significantly positive correlations with work time and performance. Long-term work stress may affect the health and life satisfaction of doctors, resulting in occupational burnout (7). Work stressors in nurses are heavy workload, the role conflict, negative experience from the event, the work task, job category, social support, and traumatic events (8–9). Anxious civil servants feel awful work stress. Job categories determine different stress handling modes and response intensities. Heavy work stress significantly affects the occupational burnout of individuals (10). Work stress and occupational burnout influence the working efficiency of nurses in intensive care units and thereby influence the satisfaction of patients and decrease nursing quality (11). Therefore, analyzing correlations can be conducive to the stress-strength intervention of civil servants. It can also relieve the problems of psychological health of civil servants brought about by political and institutional reforms such as work stressors, work stress responses, SWB of civil servants, predictive effects of work stressors and stress responses on SWB.

The present study discusses two problems: First, what is the current status and difference of work stressors of civil servants? Second, what is the effect of work stressors and work stress responses of civil servants on SWB? To address the two problems, this study attempts to discuss the current status and differences in work stressors of civil servants and correlations among work stressors, work stress responses, and SWB through a t-test, one-way analysis of variance (ANOVA), correlation analysis, and multiple regression analysis. Conclusions are expected to provide references for the stress-strength intervention of civil servants.

## Methods

### Research tools

#### Work stressors questionnaire of civil servants

The work stressors in questionnaire of civil servants was compiled by Feng and Shi (12). The results of this questionnaire are reliable. The questionnaire covered seven dimensions, namely, superiors, interpersonal relationship, job responsibility, work task, work particularity, perfectionism, and career prospect. The Cronbach’s α of this questionnaire was 0.728.

#### Work Stress responses questionnaire

Considering the research topic, concept, and adaptation of existing questionnaires, this kind of questionnaire was modified in the present study. We revised Work pressure questionnaire of teachers compiled by Shi and Cheng (13). The revised questionnaire with 10 items covers physiological and psychological responses. The Cronbach’s α of this questionnaire was 0.883.

SWB Scale for Chinese Citizens used the simplified edition, compiled by Xing (14). This scale is formed on the basis of relevant scales in China and abroad, such as the SWB Scale for Chinese Citizens, General Satisfaction with Life Scale, Domain Satisfaction with Life Scale, and Single-item Self-report SWB Scale. This scale covers 20 items and 10 dimensions, namely, contentment experience, psychological health experience, social confidence experience, growth progress experience, target value experience, self-acceptance experience, physical health experience, mental balance experience, interpersonal adaptation experience, and family atmosphere experience. The Cronbach’s α of this questionnaire was 0.731.

In addition, the data process and analysis were accomplished by SPSS 18.0 (Chicago, IL, USA) and Amos 21.0 statistical analysis software to analyze the test results of respondents on work stressors, work stress responses, and SWB. Current status and differences in work stressors in civil servants and correlations among work stressors, responses of work stress, and SWB were explored by a *t*-test, one-way ANOVA, a correlation analysis, and analysis of multivariate regression.

#### Data sources

Civil servants’ responses were randomly collected from Wuhu, Hefei, Chaohu, Nanjing, Guangzhou, and Shanghai from May to June 2018. A total of 912 questionnaires were distributed, and 868 valid questionnaires were collected, excluding incomplete questionnaires. Accordingly, the proportion of effective recovery is 95.1%. Meanwhile, six civil servants from different units in Hangzhou and Shaoxing were interviewed. Valid questionnaire data and interview records were also reviewed. The characteristic distribution of the research samples is depicted in Table 1.

**Table 1:** Descriptive statistics of the samples

***Variable***		***N***	***Ratio (%)***
Gender	Male	551	63.1
	Female	323	36.9
Age (yr)	18–30	313	35.9
	31–40	345	39.6
	41–50	189	21.7
	>51	24	2.8
Marital status	Single	251	28.7
	Married	603	69.0
	Divorced	16	1.8
	Widowed	4	0.5
Work years	Less than one year	153	17.5
	1–3 years	234	26.8
	3–10 years	226	25.8
	Over 10 years	261	29.9
Education	High school	40	4.6
	College	244	27.9
	Undergraduate	540	61.8
	Post-graduate	50	5.7
Position	General staff	647	75.0
	Deputy staff	179	20.5
	Principle staff	48	5.5

## Results

### General situations and difference of work stressors of civil servants

The general situation and difference of work stressors of respondents were analyzed in accordance with the scores of the Work Stressors Scale of Civil Servants. The results are illustrated in Tables 2–7.

**Table 2:** Descriptive statistics of work stressors on total and gender

***Factor***	***Score***	***Male (n = 548)***	***Female (n = 320)***	***t-test***
Superiors	26.12 ± 6.92	26.80 ± 6.73	24.88 ± 7.08	3.846***
Job responsibility	6.65 ± 1.79	6.88 ± 1.71	6.21 ± 1.85	5.185***
Interpersonal relationship	12.72 ± 4.27	13.21 ± 4.23	11.83 ± 4.20	4.581***
Work task	7.22 ± 1.98	7.25 ± 1.99	7.17 ± 1.97	0.527
Work particularity	8.14 ± 1.72	8.42 ± 1.71	7.61 ± 1.61	6.846***
Perfectionism	7.18 ± 1.92	7.27 ± 1.94	7.01 ± 1.87	1.886
Career prospect	7.50 ± 1.34	7.50 ± 1.33	7.49 ± 1.38	0.105
Work stressors	75.53 ± 13.76	77.34 ± 13.35	72.21 ± 13.89	5.212***

* *P*<0.05,

** *P*<0.01,

*** *P*<0.001

**Table 3:** Descriptive statistics of work stressors on age

***Variable***	***18–30***	***31–40***	***41–50***	***Over 51***	***F***
Superiors	24.62 ± 6.59	26.24 ± 6.25	27.17 ± 7.95	34.50 ± 2.02	18.957***
Job responsibility	6.27 ± 1.90	6.83 ± 1.70	6.83 ± 1.73	7.17 ± 1.37	6.855***
Interpersonal relationship	11.42 ± 3.90	12.91 ± 3.89	13.83 ± 4.84	17.17 ± 3.09	23.917***
Work task	7.22 ± 2.05	7.27 ± 1.90	7.21 ± 2.04	6.67 ± 1.93	0.688
Work particularity	7.97 ± 1.50	8.34 ± 1.84	8.00 ± 1.78	8.33 ± 1.74	2.962*
Perfectionism	7.18 ± 1.60	7.15 ± 2.13	7.06 ± 1.96	8.50 ± 1.64	4.077**
Career prospect	7.07 ± 1.13	7.37 ± 1.30	8.32 ± 1.40	8.17 ± 0.92	41.470***
Work stressors	71.75 ± 12.79	76.10 ± 12.58	78.43 ± 15.81	90.50 ± 4.60	21.196***

* *P*<0.05,

** *P*<0.01,

*** *P*<0.001

**Table 4:** Descriptive statistics of work stressors on marital status

***Variable***	***Unmarried***	***Married***	***Divorced***	***Widowed***	***F***
Superiors	24.95 ± 6.86	26.55 ± 6.73	24.75±11.32	35.00±0.00	5.436***
Job responsibility	6.33 ± 1.87	6.76 ± 1.77	6.75 ± 0.45	7.00 ± 0.00	3.235*
Interpersonal relationship	12.04 ± 4.11	12.86 ± 4.26	15.50 ± 4.16	20.00 ± 0.00	8.542***
Work task	7.14 ± 2.17	7.30 ± 1.88	5.50 ± 2.58	7.00 ± 0.00	4.533**
Work particularity	8.09 ± 1.46	8.15 ± 1.83	8.50 ± 1.15	8.00 ± 0.00	0.314
Perfectionism	7.39 ± 1.51	7.09 ± 2.04	7.00 ± 2.53	9.00 ± 0.00	2.539
Career prospect	6.96 ± 1.16	7.67 ± 1.34	8.25 ± 1.98	9.00 ± 0.00	19.921***
Work stressors	72.90 ± 12.89	76.38 ± 13.64	76.25 ± 22.54	95.00 ± 0.00	6.355***

* *P*<0.05,

** *P*<0.01,

*** *P*<0.001

**Table 5:** Descriptive statistics of work stressors on work years

***Variable***	***Less than 1 year***	***Within 1–3 years***	***Within 3–10 years***	***Over 10 years***	***F***
Superiors	22.53 ± 6.39	25.06 ± 7.08	26.45 ± 5.87	28.82 ± 6.81	31.802***
Job responsibility	6.55 ± 1.79	6.40 ± 2.01	6.75 ± 1.86	6.82 ± 1.49	2.564
Interpersonal relationship	10.37 ± 2.83	11.66 ± 3.77	13.00 ± 4.14	14.72 ± 4.50	45.325***
Work task	7.29 ± 2.40	7.17 ± 1.93	7.38 ± 1.80	7.09 ± 1.90	0.923
Work particularity	8.13 ± 1.60	7.66 ± 1.72	8.41 ± 1.73	8.29 ± 1.71	8.222***
Perfectionism	6.71 ± 1.63	6.64 ± 1.81	7.14 ± 1.92	7.92 ± 1.94	23.284***
Career prospect	7.08 ± 0.81	7.21 ± 1.35	7.43 ± 1.51	8.05 ± 1.26	24.728***
Work stressors	68.66 ± 11.27	71.79 ± 13.97	76.55 ± 11.94	81.71± 13.59	40.780***

* *P* < 0.05,

** *P* < 0.01,

*** *P* < 0.001

**Table 6:** Descriptive statistics of work stressors on educational background

***Variable***	***High school***	***College***	***Undergraduate***	***Postgraduate***	***F***
Superiors	29.00 ± 8.18	28.69 ± 5.85	24.94 ± 7.05	23.27 ± 4.18	22.688***
Job responsibility	7.40 ± 1.65	6.93 ± 1.62	6.50 ± 1.81	6.09 ± 2.13	7.217***
Interpersonal relationship	15.00 ± 3.07	14.48 ± 4.07	11.76 ± 4.15	12.27 ± 3.88	29.122***
Work task	7.00 ± 2.35	7.13 ± 1.62	7.22 ± 2.09	7.91 ± 2.04	2.107
Work particularity	8.00 ± 1.50	8.23 ± 1.62	8.16 ± 1.76	7.45 ± 1.90	2.677*
Perfectionism	7.30 ± 2.44	7.66 ± 1.87	6.95 ± 1.92	7.18 ± 1.13	7.794**
Career prospect	7.70 ± 0.91	7.85 ± 1.39	7.34 ± 1.37	7.27 ± 0.45	9.051**
Work stressors	81.40 ± 15.90	80.97 ± 12.17	72.87 ± 13.55	71.45 ± 11.96	24.833***

* *P* < 0.05,

** *P* < 0.01,

*** *P* < 0.001

**Table 7:** Descriptive statistics of work stressors on position

***Variable***	***General staff***	***Deputy staff***	***Principal staff***	***F***
Superiors	26.00 ± 6.91	25.98 ± 7.15	28.33 ± 5.77	2.609
Job responsibility	6.54 ± 1.68	6.75 ± 2.07	7.67 ± 1.72	9.438***
Interpersonal relationship	12.75 ± 4.34	12.25 ± 4.17	14.08 ± 3.34	3.555*
Work task	7.22 ± 1.97	7.20 ± 1.92	7.25 ± 2.41	0.012
Work particularity	8.09 ± 1.68	8.18 ± 1.83	8.58 ± 1.77	1.914
Perfectionism	7.19 ± 1.90	7.02 ± 2.03	7.58 ± 1.67	1.664
Career prospect	7.47 ± 1.34	7.68 ± 1.30	7.17 ± 1.48	3.213*
Work stressors	75.26 ± 13.56	75.07 ± 15.33	80.67 ± 8.42	3.584*

* *P* < 0.05,

** *P* < 0.01,

*** *P* < 0.001

Table 2 reveals that the general score of work stressors of civil servants was 75.53 ± 13.76. Several factors in different dimensions of work stressors had various scores. Specifically, superiors achieved the highest score (26.12 ± 6.92), followed by interpersonal relationship (12.72 ± 4.27). Scores on work task (7.22 ± 1.98) and perfectionism (7.18 ± 1.92) were similar. The lowest score was achieved by job responsibility (6.65 ± 1.79).

The one-way ANOVA results on work stressors scores of different ages of civil servants are presented in Table 3. The total scores in different dimensions (superiors, interpersonal relationship, work particularity, career prospect, perfectionism, job responsibility, and work stressors) had significantly disparity on different ages of civil servants (*P <* 0.001).

The effects of marital status on the scores of work stressors of civil servants were analyzed through one-way ANOVA (Table 4). Significant differences were found between married and unmarried civil servants in the scores of superiors, job responsibility, interpersonal relationship, and career prospect and total scores (*P <* 0.05).

The one-way ANOVA analysis results of the effects of working years on work stressors of civil servants are depicted in Table 5. With the increase of working years of civil servants, the level of stressors of work in civil servants also increased. Significant differences on the scores of superiors, interpersonal relationship, work particularity, perfectionism, and career prospect and total scores were observed with the increase of working years (*P <* 0.001).

The one-way ANOVA analysis results of the effects of educational background on work stressors of civil servants are illustrated in Table 6. Educational background could significantly act upon scores of superiors, job responsibility, interpersonal relationship, work particularity, perfectionism, and career prospect and total scores (*P <* 0.01). Table 7 reveals that except for career prospect, the scores of principal civil servants in other dimensions and general sources are higher than those of deputy positions and common positions. According to the one-way ANOVA results of work stressors scores of civil servants in different posts, significant differences were only observed on job responsibility, interpersonal relationship, and career prospect and total scores (*P <* 0.05).

### Correlations among work stressors, work stress responses, and SWB of civil servants

The correlations among work stressors, work stress responses, and SWB of civil servants were analyzed in accordance with the work stressors, work stress responses, and SWB Scale results, respectively (Tables 8 and 9).

**Table 8:** Correlations between work stressors and work stress responses

***Variable***	***Work Stress responses***	***Physiological response***	***Psychological response***
Superiors	0.504**	0.335**	0.548**
Job responsibility	0.125**	0.043	0.163**
Interpersonal relationship	0.400**	0.245**	0.448**
Work task	0.096**	0.036	0.123**
Work particularity	0.228**	0.178**	0.230**
Perfectionism	0.335**	0.222**	0.364**
Career prospect	0.337**	0.279**	0.330**
Work stressors	0.515**	0.335**	0.565**

* *P* < 0.05,

** *P* < 0.01,

*** *P* < 0.001

**Table 9:** Correlations among work stressors, work stress responses, and subjective well-being

***Variable***	***Work stressors***	***Work Stress responses***	***Subjective Well-being***
Work stressors	1		
Work Stress responses	0.515**	1	
Subjective Well-being	−0.073*	−0.114**	1

* *P* < 0.05,

** *P* < 0.01,

*** *P* < 0.001

In Table 8, the total score of the work stressors Scale is significantly positively correlated with the total score of the work stress responses, physiological response, and psychological response scales. The total scores of the work stress response and psychological response scales were significantly correlated with the different dimensions of work stressors (*P <* 0.01).

The physiological response was significantly positively correlated with superiors, interpersonal relationship, work particularity, perfectionism, and career prospect (*P <* 0.01).

Table 9 reveals a significantly positive correlation between the total score of the work stressors and work stress responses (*P* < 0.01), showing a correlation coefficient of 0.515. A significantly negative correlation existed between the total scores of the work stressors and SWB (*P* < 0.05), with a correlation coefficient of −0.073. A significantly negative correlation existed between the total score of the work stress responses and SWB (*P* < 0.01), with a correlation coefficient of −0.114.

### Regression analysis results of work stressors, work stress responses, and SWB of civil servants

A stepwise multiple regression analysis was carried out to further reveal the correlations among work stressors, work stress responses, and SWB. In the regression analysis, SWB was used as the observed variable (dependent variable), whereas seven dimensions of work stressors and two dimensions of work stress responses were used as predictive variables (independent variables) (Table 10). Only four dimensions of work stressors (namely, work particularity, work task, perfectionism, and job responsibility) were included in the regression equation, finding *R^2^* = 0.093 and adjusted *R^2^* = 0.085 (*P <* 0.01). This result demonstrates that the regression equation was generally significant, and four dimensions explain 8.5% of the variation. Two dimensions of work stress responses were involved in the regression equation, revealing *R^2^* = 0.007 and 0.020 (*P* < 0.05, 0.01). This finding indicates that the regression equation is generally significant. The two factors account for 0.7% and 2% of the total variation respectively.

**Table 10:** Effects of subjective well-being on work stressors and work stress responses

***Dependent variable***	***Independent variables***	***Beta***	***T***	***Sig.***
Subjective Well-being	Work particularity	0.191	5.645	0.000
	Job responsibility	−0.172	−5.182	0.000
	Perfectionism	0.104	2.945	0.003
	Work Task	−0.112	−3.207	0.001
	Psychological Response	−0.082	−2.383	0.017
	Physiological Response	−0.158	−3.411	0.001

## Discussions

Civil servants are apprehensive on career prospect, which is caused by fierce vocational competition, conflicts between career expectations and practices, occupational promotion, interpersonal relationships, supervision by social public opinions, cognitive bias and error of the public society, single management mode of existing systems, difficulty in objective measurement and assessment, and conflicts between expected effects and limited actual effects of the reward and punishment mechanism (15–17).

With the continuous expansion of the functions of government sectors, the workload and working content of civil servants have significantly changed. In particular, faced with the new situation of strengthening self-discipline within the Communist Party of China, severe punishment is applied for the dereliction of duty and mistakes of civil servants in recent years (18). Therefore, work tasks and job responsibilities become the major stressors of work for civil servants. Based on the analysis of the results in Table 2, male civil servants generally feel heavy work burden than female ones, which conforms to the results of other related surveys. Table 3 demonstrates that scores of work stressors for civil servants aged from 31 to 40 and 41 to 50 are relatively high. Civil servants of this age are facing a critical point of career development and promotion and are expected to support their aged parents and young children. Therefore, they often bear tremendous psychological pressure. Table 4 reveals that widowed civil servants face high-intensity working pressure than other civil servants. In Table 5, the level of work stressors of civil servants is raised with the increase of working age. Civil servants who have worked for more than 10 years receive the highest score. Table 6 reveals that with the level of perfection requirement in the civil servant management system, the employment standards of government sectors have also improved and civil servants with a higher educational background have stronger competence. Table 7 illustrates that civil servants in prominent position assume high responsibilities and thereby facing greater work pressure.

Based on Tables 8 and 9, the general scores of the work stressors are significantly positively correlated with the general scores of the work stress responses and physiological response and psychological response. The work stressors in civil servants are significantly negatively correlated with the total score of SWB. The work stress response of civil servants is significantly negatively correlated with the total scores of SWB. These correlations reflect that civil servants facing stronger work stress make strong stress responses and have a low SWB level. The work stressors and work stress responses of civil servants both have an effect on SWB. Table 10 demonstrates that work particularity and perfectionism have positive predictive effects on SWB. However, work task, job responsibility, psychological response, and physiological response have negative predictive effects on SWB.

Therefore, government departments should devote themselves to the construction of civil servants’ mental health service system to meet the needs of the new era, and the mental health of Chinese civil servants should be taken as an important part in promoting the construction of political ecological civilization. Hobfoll argued that work stress will consume the beneficial resources of individuals and finally cause negative consequences of stress. Individuals possess enough resources to relieve and offset such wastes, preventing negative consequences (19). The establishment of social support, exercises and fitness are all conducive to relieve work stress and increase the level of psychological health among civil servants (20–21).

As the key component of microcosmic management, the government can positively carry out occupational psychological service and intervention with civil servants from the professional perspective of the human resource management of public sectors, help civil servants adapt to the changes of working roles, adjust occupational emotion in a timely manner, relieve work stress, and avoid risks at work.

## Conclusion

SWB can be accurately predicted by stressors of work pressure and responses. Further analysis of the regulatory effect can have influence on the feasibility of intervention for work stress of civil servants. Therefore, in-depth associated studies can be carried out in the future. In addition, survey respondents mainly consist of urban civil servants in service but involve few civil servants in the township, thus influencing the representativeness of the samples. Accordingly, future studies can expand the scope of respondents.

## Ethical considerations

Ethical issues (Including plagiarism, informed consent, misconduct, data fabrication and/or falsification, double publication and/or submission, redundancy, etc.) have been completely observed by the authors.
